# The endothelial function biomarker soluble E‐selectin is associated with nonalcoholic fatty liver disease

**DOI:** 10.1111/liv.14384

**Published:** 2020-01-29

**Authors:** Nynke Simons, Mitchell Bijnen, Kristiaan A. M. Wouters, Sander S. Rensen, Joline W. J. Beulens, Marleen M. J. van Greevenbroek, Leen M. ’t Hart, Jan Willem M. Greve, Carla J. H. van der Kallen, Nicolaas C. Schaper, Casper G. Schalkwijk, Coen D. A. Stehouwer, Martijn C. G. J. Brouwers

**Affiliations:** ^1^ Department of Internal Medicine Division of Endocrinology and Metabolic Diseases Maastricht University Medical Center Maastricht The Netherlands; ^2^ Department of Internal Medicine Division of General Internal Medicine Laboratory for Metabolism and Vascular Medicine Maastricht University Medical Center Maastricht The Netherlands; ^3^ CARIM School for Cardiovascular Diseases Maastricht University Maastricht The Netherlands; ^4^ Department of General Surgery Maastricht University Medical Center Maastricht The Netherlands; ^5^ NUTRIM School of Nutrition and Translational Research in Metabolism Maastricht University Maastricht The Netherlands; ^6^ Department of Epidemiology and Biostatistics Amsterdam University Medical Center – location VUmc the Amsterdam Public Health Research Institute Amsterdam Amsterdam The Netherlands; ^7^ Julius Center for Health Sciences and Primary Care University Medical Center Utrecht Utrecht The Netherlands; ^8^ Department of Cell and Chemical Biology Leiden University Medical Center Leiden The Netherlands; ^9^ Department of Biomedical Data Sciences Section Molecular Epidemiology Leiden University Medical Center Leiden The Netherlands; ^10^ Department of General Surgery Zuyderland Medical Center Heerlen The Netherlands; ^11^ CAPHRI School for Public Health and Primary Care Maastricht University Maastricht The Netherlands; ^12^ Department of Internal Medicine Division of General Internal Medicine Maastricht University Medical Center Maastricht The Netherlands

**Keywords:** endothelium, E‐selectin, genetic epidemiology, nonalcoholic fatty liver disease, translational research

## Abstract

**Background & Aims:**

Plasma soluble E‐selectin (sE‐selectin) is a frequently used biomarker of systemic endothelial dysfunction. The present study explored the relationship between nonalcoholic fatty liver disease (NAFLD) and plasma sE‐selectin levels.

**Methods:**

Expression of E‐selectin in liver, visceral adipose tissue (VAT) and muscle was studied in relation to plasma sE‐selectin in severely obese individuals (n = 74). The course of hepatic E‐selectin expression in relation to hepatic steatosis and inflammation was examined in C57BL/6J LDLR^−/−^ mice on a Western‐type diet. The relationship between biomarkers of NAFLD, that is, plasma aminotransferase (ALT) and NAFLD susceptibility genes (rs738409 [*PNPLA3*] and rs1260326 [*GCKR*]), and plasma sE‐selectin was studied in the combined CODAM (n = 571) and Hoorn (n = 694) studies.

**Results:**

E‐selectin expression in liver, not VAT or muscle, was associated with plasma sE‐selectin in severely obese individuals (β = 0.26; 95% CI: 0.05‐0.47). NAFLD severity was associated with hepatic E‐selectin expression (*P* = .02) and plasma sE‐selectin (*P* = .003). LDLR^−/−^ mice on a Western‐type diet displayed increased hepatic E‐selectin expression that followed the same course as hepatic inflammation, but not steatosis. In the CODAM study, plasma ALT was associated with plasma sE‐selectin, independent of potential confounders (β = 0.25; 95% CI: 0.16‐0.34). Both rs738409 and rs1260326 were associated with higher plasma sE‐selectin in the combined CODAM and Hoorn studies (*P* = .01 and *P* = .004 respectively).

**Conclusions:**

NAFLD and related markers are associated with higher expression of hepatic E‐selectin and higher levels of plasma sE‐selectin. Further studies are required to investigate the role of E‐selectin in the pathogenesis of NAFLD and the applicability of sE‐selectin as a plasma biomarker of NAFLD/NASH.

**Abbreviations**
AFLD: alcoholic fatty liver disease
CODAM: Cohort on Diabetes and Atherosclerosis Maastricht
CVD: cardiovascular disease
GCKR: glucokinase regulatory protein
IGM: impaired glucose metabolism
NAFLD: nonalcoholic fatty liver disease
NAS: NAFLD activity score
NASH: nonalcoholic steatohepatitis
NGM: normal glucose metabolism
PNPLA3: patatin‐like phospholipase domain‐containing protein 3
sE‐selectin: soluble E‐selectin
sVCAM‐1: soluble vascular cell adhesion molecule‐1
T2DM: type 2 diabetes
VAT: visceral adipose tissue

**Key points**

- The pathogenesis of nonalcoholic fatty liver disease (NAFLD) is multifactorial. Liver sinusoidal endothelial cells have been suggested to play a role.
- The aim of the present study was to assess the relationship between NAFLD and markers of endothelial activation (ie expression and plasma levels of E‐selectin) in mice, severely obese individuals undergoing bariatric surgery and the general population.
- NAFLD severity is associated with hepatic E‐selectin expression in mice and severely obese individuals.
- E‐selectin expression in liver, but not in other organs, is associated with plasma sE‐selectin levels in severely obese individuals.
- NAFLD susceptibility genes, that is, *PNPLA3 and GCKR*, and plasma ALT levels are associated with plasma sE‐selectin levels, independent of potential confounding factors.
- These findings favour further study to elucidate the role of E‐selectin in the pathogenesis of NAFLD and the applicability of sE‐selectin as a plasma biomarker of NAFLD/NASH.

## 1 INTRODUCTION

Nonalcoholic fatty liver disease (NAFLD) is emerging as the principal cause of end‐stage liver disease requiring transplantation.^1^ The pathogenesis is complex and involves genetic, inflammatory, (gut) microbiotic, metabolic, nutritional and lifestyle factors.^2^

Recent studies have implicated a role for liver sinusoidal endothelial cells in the pathogenesis of NAFLD. Liver sinusoidal endothelial dysfunction favours the development of steatosis and recruitment of inflammatory cells.^3^ This premise is largely based on animal studies, given the difficulties of acquiring human liver tissue to conduct endothelial function tests.

Plasma soluble E‐selectin (sE‐selectin) and vascular cell adhesion molecule‐1 (sVCAM‐1) have often been used as biomarkers of *systemic* endothelial activation.^4^, ^5^, ^6^ Of interest, we and others have previously shown that NAFLD is a significant determinant of plasma endothelial biomarkers, in particular sE‐selectin.^7^, ^8^ Furthermore, it has been shown that *hepatic* E‐selectin is actively involved in hepatic neutrophil infiltration in mice exposed to chronic plus binge ethanol feeding.^9^

The aim of the present study was, therefore, to study the relationship between NAFLD and sE‐selectin in more detail. We aimed to assess (a) the relationship between NAFLD and hepatic E‐selectin expression, (b) the relationship between hepatic E‐selectin expression and plasma sE‐selectin levels and (c) the association between NAFLD and plasma sE‐selectin levels, independent of potential confounding factors. For this, a diverse set of studies was used, ranging from liver expression data in severely obese individuals and a mouse model of nonalcoholic steatohepatitis (NASH), to (genetic) epidemiology in two large cohorts.

## 2 MATERIALS AND METHODS

### 2.1 Liver biopsies in severely obese individuals undergoing bariatric surgery

Details of this study have been described elsewhere.^10^, ^11^ Briefly, liver, muscle and visceral adipose tissue (VAT) biopsies were collected from severely obese individuals (BMI > 40 kg/m^2^, n = 74) undergoing elective bariatric surgery at the department of General Surgery of Maastricht University Medical Center (Maastricht, The Netherlands) between 2006 and 2009. Exclusion criteria were inflammatory or degenerative diseases, use of anti‐inflammatory drugs or alcohol intake >10 g/d. All participants gave informed consent. The study was performed according to the Declaration of Helsinki^12^ and approved by the Medical Ethical Committee of Maastricht University Medical Center (Maastricht, The Netherlands). Liver biopsy samples were fixed in formalin and stained with Haematoxylin and Eosin (H&E), periodic acid‐Schiff with diastase and Masson's trichrome, and subsequently scored for the presence of steatosis, inflammation (ie lobular and portal inflammation and hepatocellular ballooning) and fibrosis by an experienced pathologist blinded for the study context and outcomes. Individuals were divided into three groups according to the NAFLD activity score (NAS): ≤2 (no NASH), 3‐4 (probable NASH) and ≥5 (definite NASH).^13^ E‐selectin and VCAM mRNA expression in liver, VAT and muscle were determined using Illumina HumanHT12 Bead‐Chips (Illumina) and an Illumina BeadArray Reader. Plasma was drawn on the morning of the surgery after an overnight fast of 8 hours for measurement of various metabolic traits via routine clinical chemistry. Plasma levels of sE‐selectin and sVCAM were determined using a Diaclone ELISA kit (Diaclone SAS). Histology, expression and plasma data were available in subsets of the study population; the number of individuals, therefore, differ between analyses.

### 2.2 LDLR^−/−^ mice fed a Western‐type diet

Female (16‐ to 18‐week old) C57BL/6 LDLR^−/−^ mice were fed a chow or Western‐type diet (21% milk butter, 0,2% cholesterol, 46% carbohydrates [of which 40,5% sucrose], and 17% casein; SDSdiets #824171) for 16 hours, 48 hours, 72 hours, 1 week or 3 weeks and were subsequently sacrificed via CO_2_/O_2_ inhalation. This Western‐type diet is known to rapidly induce hepatic steatosis and inflammation in LDLR^−/−^ mice and has been used as model for NASH in numerous studies.^14^, ^15^, ^16^ Liver paraffin sections were stained for H&E and photographed using a Zeiss microscope (Axioskop 40) with a Jenoptik camera and Progress Capture Pro 2.8.8 software package. Hepatic inflammation (immune cell count and clustering, and hepatocyte injury) and steatosis (lipid droplets) were scored semi‐quantitatively (0‐4) in a blinded fashion by two independent experienced researchers. The average score was used for statistical analysis. RNA was isolated from the liver using Trizol reagent (Ambion). Next, E‐selectin and TNF gene expression were determined using IQ SensiMix SYBR master mix (Bioline) on a CFX96 Touch with CFX manager software (Biorad). The geometric mean of Cyclophillin and Beta2‐microglobulin was used as reference (see Table S1 for primer sequences) and the ΔΔCT method was applied to calculate the gene expression levels.^17^ Plasma sE‐selectin levels were determined using a DuoSet ELISA kit (R&D Systems). All experiments were approved by the Animal Experiments Committee of Maastricht University (Maastricht, The Netherlands) and in compliance with the relevant guidelines from the Directive 2010/63/EU of the European Parliament on the protection of animals used for scientific purposes.

### 2.3 The combined Hoorn and CODAM studies

The Hoorn and Cohort on Diabetes and Atherosclerosis Maastricht (CODAM) studies are observational, prospective cohort studies on determinants and (cardiovascular) complications of type 2 diabetes mellitus (T2DM). The original Hoorn study was executed in 2484 randomly selected residents of Hoorn between 1989 and 1992.^18^ Between 2000 and 2001, follow‐up measurements were conducted in a subset (n = 822) of participants,^18^ which were used for the current study. The CODAM study was performed in 574 participants of the Dutch Monitoring Project for Cardiovascular Diseases (MORGEN) and its predecessor with an elevated risk of T2DM.^19^, ^20^ All participants underwent a standard 75‐g oral glucose tolerance test to determine glucose metabolism state, that is, normal glucose metabolism (NGM), impaired glucose metabolism (IGM) (comprising both impaired fasting glucose and impaired glucose tolerance) and T2DM. All participants provided informed consent. The Hoorn and CODAM studies were performed according to the Declaration of Helsinki^12^ and approved by the local Medical Ethical Committees. As the protocols of both studies were similar, data were combined for statistical analyses, as has been done before.^21^, ^22^

In the combined cohorts, 1286 individuals were available for genotyping. Genotyping of *GCKR* (rs1260326) and *PNPLA3* (rs738409) was performed with validated Invitrogen TaqMan assays (Thermo Fisher Scientific). In CODAM, *GCKR* (rs1260326) was genotyped as part of a genome‐wide association study array, using a HumanOmniExpress BeadChip (Illumina).^21^ Genotyping of rs1260326 and rs738409 was successful in 1265 individuals. Both variants were in Hardy‐Weinberg equilibrium (*P* = .93 for rs1260326 [minor allele frequency: 38.5%] and *P* = .16 for rs738409 [minor allele frequency: 24.2%] in individuals with NGM). Plasma was drawn after an overnight fast for measurement of among others alanine aminotransferase (ALT), triglycerides, sE‐selectin and sVCAM levels, as described before.^18^, ^20^ Plasma levels of sE‐selectin and sVCAM were determined using a MSD multiplex assay (Meso Scale Diagnostics), which is comparable to single‐biomarker methods, such as ELISA, but could result in a different absolute concentration.^23^ In the Hoorn study, plasma ALT levels were only determined at the baseline visit (1989‐1992), not during the follow‐up measurements (2000‐2001) that were used for the present study. The relationships of plasma ALT with sE‐selectin and sVCAM levels were therefore studied in participants of the CODAM study only. Since genotype is time‐independent, the associations of rs1260326 and rs738409 with plasma ALT (and triglycerides, sE‐selectin and sVCAM) were studied in the combined Hoorn and CODAM studies.

### 2.4 Statistical analyses

Data are presented as percentage of total, mean ± SD or median (interquartile range). Non‐normally distributed variables were log transformed before further analyses. Differences between groups or associations between variables were analysed using one‐way ANOVA or linear/logistic regression, respectively, with adjustments for age, sex, and – if applicable – cohort (ie the Hoorn and CODAM studies). For readability purposes, figure data are presented after adjustment for age and sex (and cohort) in which the residuals are normalized to the average age and sex (and cohort) of that study population.

Multivariable linear regression was used to study the associations between plasma ALT and sE‐selectin and sVCAM levels, independent of potential confounding factors, that is, BMI, smoking, alcohol intake, plasma lipids (ie total cholesterol and HDL‐cholesterol), systolic blood pressure, HbA1c, use of glucose‐lowering medication, use of lipid‐modifying medication, inflammatory markers (ie C‐reactive protein, amyloid A, interleukin‐6, interleukin‐8 and tumour necrosis factor α) and history of cardiovascular disease. Finally, the associations between the NAFLD susceptibility genes *PNPLA3* (rs738409) or *GCKR* (rs1260326) and plasma ALT, triglycerides, sE‐selectin and sVCAM levels were analysed under the assumption of an additive mode of inheritance, based on previous reports.^24^, ^25^, ^26^ Results were considered statistically significant at *P* < .05. All statistical analyses were carried out by the IBM Statistical Package of Social Sciences (SPSS) version 23 for Windows (SPSS Inc).

## 3 RESULTS

### 3.1 NAFLD histological stage is associated with hepatic E‐selectin mRNA expression in severely obese individuals

Hepatic E‐selectin and VCAM mRNA expression patterns were studied in severely obese individuals undergoing bariatric surgery (see Table 1 for characteristics). Hepatic E‐selectin mRNA expression increased with NAFLD severity from 7.05 ± 0.22 (NAS ≤ 2), to 7.12 ± 0.26 (NAS 3‐4) and to 7.24 ± 0.23 (NAS ≥ 5) (*P* for trend = .02), whereas an opposite trend was observed for hepatic VCAM mRNA expression (7.92 ± 0.21 [NAS ≤ 2], 7.77 ± 0.28 [NAS 3‐4] and 7.80 ± 0.21 [NAS ≥ 5], *P* for trend = .09). Hepatic E‐selectin mRNA expression was associated with steatosis and fibrosis grade (*P* = .004 and *P* = .002 respectively).

**Table 1:** General characteristics of severely obese individuals undergoing bariatric surgery and participants of the combined CODAM and Hoorn studies

	Bariatric surgery (n = 74)	CODAM/Hoorn (n = 1265)
Male/female	23/51	562/703
Age (y)	44.5 ± 9.8	64.6 ± 8.4
BMI (kg/m^2^)	44.4 (39.0‐50.2)	28.1 ± 4.2
Waist‐hip ratio	0.97 (0.89‐1.11)	0.94 ± 0.09
T2DM (%)	34.9%	33.2%
Glucose (mmol/L)	5.8 (5.3‐7.2)	5.9 (5.3‐6.8)
HbA1c (%)	6.2 (5.6‐6.8)	5.9 (5.6‐6.3)
Insulin (pmol/L)	111 (69‐160)	66 (46‐100)
Glucose‐lowering medication (% yes)	25.5%^b^	10.3%
Total cholesterol (mmol/L)	5.0 ± 1.1	5.5 ± 1.0
LDL cholesterol (mmol/L)	3.2 ± 1.0	3.4 ± 0.9
HDL cholesterol (mmol/L)	0.9 (0.7‐1.1)	1.2 (1.0‐1.5)
Triglycerides (mmol/L)	1.75 (1.23‐2.63)	1.4 (1.0‐1.9)
Lipid‐modifying medication (% yes)	25.5%^b^	17.5%
ALT (U/L)	22 (17‐31)	22 (17‐28)^a^
AST (U/L)	23 (16‐31)	20 (16‐24)^a^
Alcohol (g/d)	Nd	6.7 (0.7‐20.3)
Systolic blood pressure (mmHg)	138 ± 18	142 ± 20
Diastolic blood pressure (mmHg)	81 ± 11	83 ± 10
Antihypertensive medication (% yes)	45.5%^b^	38.9%
Smoking (% yes)	30.8%^b^	18.1%
History of cardiovascular disease (% yes)	13.8%^b^	42.4%

Note

Data are expressed as percentage of total, mean ± SD or median (interquartile range).

Abbreviations: ALT, alanine aminotransferase; AST, aspartate aminotransferase; BMI, body mass index; CODAM, Cohort on Diabetes and Atherosclerosis Maastricht; HbA1c, haemoglobin A1c; HDL, high‐density lipoprotein; IGM, impaired glucose metabolism; LDL, low‐density lipoprotein; nd, not determined; NGM, normal glucose metabolism; T2DM, type 2 diabetes.

a Presented for participants of the CODAM study only (n = 571). In the Hoorn study, plasma ALT levels have been measured at another visit, see methods section.

b 20%‐50% of total study population (n = 74) variable unknown.

### 3.2 Hepatic E‐selectin mRNA expression coincides with hepatic inflammation in LDLR^−/−^ mice fed a Western‐type diet

**Figure 1:**
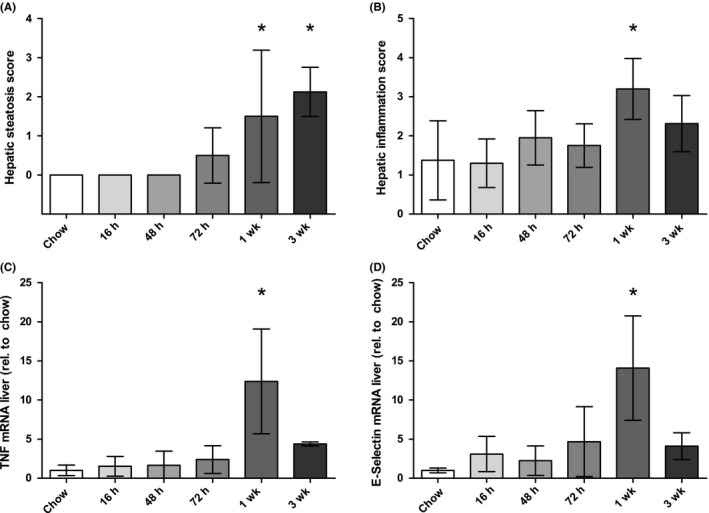
Liver steatosis score (A), inflammation score (B), hepatic TNF mRNA expression (C) and hepatic E‐selectin mRNA expression (D) in LDLR^−/−^ mice fed a Western‐type diet. Time points refer to time of sacrifice, see methods section. N = 5 mice per group. Data are expressed as mean ± SD, analysed with linear regression. *P < .05 versus chow

Since steatosis and inflammation often coincide, it is difficult to assess the independent relationship of each histological stage with hepatic E‐selectin mRNA expression. We therefore determined hepatic E‐selectin mRNA expression in LDLR^−/−^ mice fed a Western‐type diet for 16 hours, 48 hours, 72 hours, 1 week and 3 weeks. We have previously shown that this model results in hepatic inflammation that precedes hepatic triglycerides accumulation,^15^ and therefore allows a disentanglement of hepatic steatosis from inflammation. Indeed, the onset of hepatic steatosis was observed after 72 hours and increased until 3 weeks on a Western‐type diet (*P* = .01 and *P* = .001 for 1 and 3 weeks versus chow diet, respectively, Figure 1A), whereas hepatic inflammation reached its peak at 1 week, but appeared to decrease at week 3 (*P* = .001 and *P* = .08 for 1 and 3 weeks versus chow diet, respectively, Figure 1B). In line with this inflammation score, hepatic TNF mRNA expression was strongly upregulated only after 1 week Western‐type diet (*P* < .001, Figure 1C). Hepatic E‐selectin mRNA expression followed a similar pattern and was only significantly different from the chow diet on week 1 (*P* = .001, Figure 1D). Of note, this was not the case for plasma sE‐selectin levels, which already increased after 72 hours and remained stable thereafter (Figure S1).

### 3.3 Hepatic E‐selectin mRNA expression is associated with plasma sE‐selectin levels in severely obese individuals

**Figure 2:**
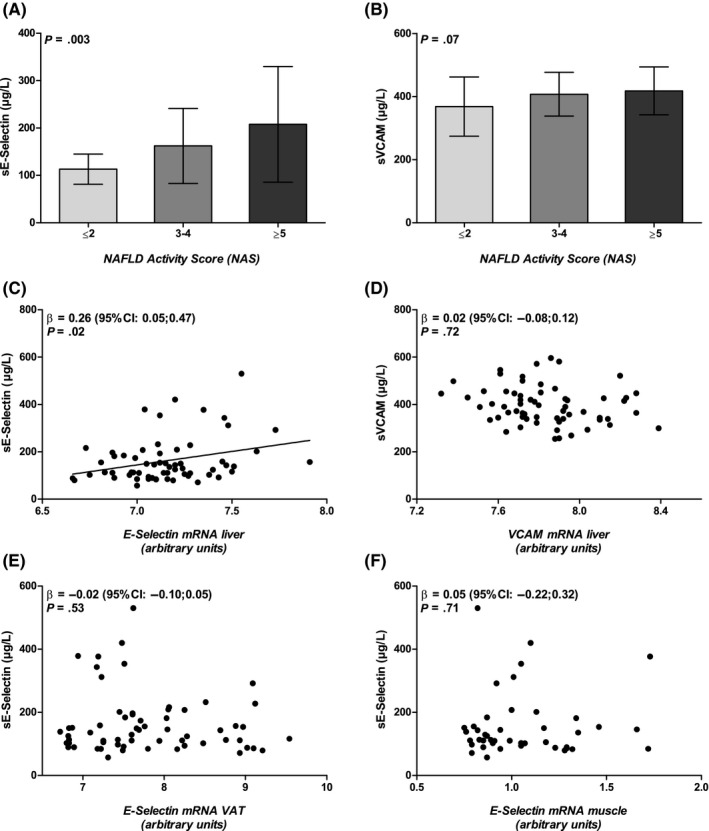
Relationship between E‐selectin mRNA expression and plasma sE‐selectin levels. Association between nonalcoholic fatty liver disease (NAFLD) severity (according to NAFLD activity score) and plasma sE‐selectin levels (A; n = 58) and sVCAM levels (B; n = 58) in severely obese individuals; E‐selectin mRNA expression in liver (C; n = 62), visceral adipose tissue (VAT) (E; n = 62) and muscle (F; n = 43) in relation to plasma sE‐selectin levels. Hepatic VCAM mRNA expression in relation to plasma sVCAM levels (D; n = 62). Data are expressed as mean (adjusted for age and sex) ± SD, analysed with one‐way ANOVA (A and B), or as individual data points (adjusted for age and sex), analysed with linear regression (C‐F)

Analogous to the hepatic mRNA expression pattern, we observed a significantly positive relationship between NAS and plasma sE‐selectin levels in severely obese individuals (*P* for trend = .003, Figure 2A). In contrast to the negative trend between NAS and hepatic VCAM mRNA expression, a positive trend was now found for the relationship between NAS and plasma sVCAM levels (*P* = .07, Figure 2B). The discriminatory ability of sE‐selectin levels to distinguish NASH (ie NAS ≥ 5) from non‐NASH (ie NAS ≤ 2) was statistically significant (area under receiver operating characteristic [ROC] curve: 0.73; 95% CI: 0.58‐0.88, Figure S2A). Similar area under the curves were observed when the NAFLD histological stages were analysed separately, except for fibrosis. Statistical significance was reached only for steatosis (Figure S2B‐E).

Hepatic E‐selectin mRNA expression was significantly associated with plasma sE‐selectin levels (unstandardized β coefficient = 0.26 [95% CI: 0.05‐0.47], Figure 2C). Such an association was not observed between hepatic VCAM mRNA expression and sVCAM levels (β = 0.02 [95% CI: −0.08 to 0.12], Figure 2D). Visceral adipose tissue and muscle E‐selectin mRNA expression patterns were not associated with plasma sE‐selectin levels (β = −0.02 [95% CI: −0.10 to 0.05], Figure 2E; and β = 0.05 [95% CI: −0.22; 0.32], Figure 2F respectively).

### 3.4 Plasma ALT levels are independently associated with plasma sE‐selectin levels in the CODAM study

Linear regression models were constructed to determine the (independent) contribution of the liver to plasma sE‐selectin levels at the population level, that is, in the CODAM study (n = 571). Plasma ALT levels were associated with plasma sE‐selectin levels after adjustment for age and sex (β = 0.35 [95% CI: 0.27‐0.42], model 1, Table 2). The strength of association decreased after subsequent adjustment for BMI, smoking behaviour and alcohol intake, but remained statistically significant (β = 0.29 [95% CI: 0.21‐0.37], model 2, Table 2). BMI, alcohol intake and smoking behaviour were all independently associated with sE‐selectin levels (although the association for smoking behaviour was lost in the final model; Table S2). Further adjustment for cardiovascular risk factors (model 3), inflammatory markers (model 4) and cardiovascular disease (model 5) did not essentially alter the relationship between plasma ALT and sE‐selectin levels (β = 0.25 [95% CI: 0.16‐0.34], model 5, Table 2). Plasma ALT levels were also independently associated with plasma sVCAM (Table 2, models 1‐5). The strength of association almost halved after adjustment for inflammatory factors (Table 2, model 4), which was mainly accounted for by TNFα (data not shown).

**Table 2:** Multivariable regression analysis for the relationship of plasma ALT levels with plasma sE‐selectin or sVCAM levels in the Cohort on Diabetes and Atherosclerosis Maastricht study

Model^a^	log plasma sE‐selectin (n = 571)			log plasma sVCAM (n = 571)		
	β	95% CI	*P* value	β	95% CI	*P* value
1	0.346	0.271;0.422	<.001	0.112	0.071;0.152	<.001
2	0.286	0.205;0.367	<.001	0.099	0.055;0.143	<.001
3	0.264	0.177;0.351	<.001	0.101	0.055;0.147	<.001
4	0.248	0.159;0.338	<.001	0.065	0.020;0.110	.005
5	0.249	0.160;0.339	<.001	0.066	0.021;0.111	.004

Note

Analysed with linear regression. Betas represent unstandardized regression coefficients.

Model 2: adjusted for model 1 + BMI, smoking and alcohol intake.

Model 3: adjusted for model 2 + cardiovascular risk factors (total cholesterol, HDL‐cholesterol, systolic blood pressure, HbA1c, use of glucose‐lowering medication, and use of lipid‐modifying medication).

Model 4: adjusted for model 3 + inflammatory markers (C‐reactive protein, amyloid A, interleukin‐6, interleukin‐8, and tumour necrosis factor α).

Model 5: adjusted for model 4 + history of cardiovascular disease.

a Model 1: adjusted for age + sex.

### 3.5 NAFLD susceptibility genes co‐segregate with plasma sE‐selectin levels in the CODAM and Hoorn studies

To further examine the relationship between NAFLD and plasma sE‐selectin levels, independent of potential confounders, we studied the association of two NAFLD susceptibility genes, that is, *PNPLA3* (rs738409) and *GCKR* (rs1260326), with plasma sE‐selectin levels in the combined CODAM and Hoorn studies (n = 1265, see Table 1 for characteristics). Previous studies have shown that both gene variants predispose to NAFLD (including NASH) and elevated liver enzymes,^26^, ^27^, ^28^ but have opposing effects on factors that are associated with *systemic* endothelial activation, that is, plasma lipids,^24^, ^29^ T2DM 30 and coronary artery disease risk^31^, ^32^ (Table S3). Indeed, positive trends were observed for the relationships of the rs1260326 T‐allele (*GCKR*) and rs738409 G‐allele (*PNPLA3*) with plasma ALT levels (β = 0.011 [95% CI: −0.004 to 0.26], *P* = .15; and β = 0.023 [95% CI: 0.005‐0.041], *P* = .01, respectively, Table 3), whereas opposing effects were found for plasma triglycerides levels (β = 0.017 [95% CI: 0.006‐0.027], *P* = .002; and β = −0.016 [95% CI: −0.029 to − 0.004], *P* = .009, respectively, Table 3). Statistically significant, positive associations were observed for both *GCKR* and *PNPLA3* with plasma sE‐selectin levels (β = 0.019 [95% CI: 0.006‐0.032], *P* = .004; and β = 0.020; [95% CI: 0.005‐0.036], *P* = .01, respectively, Table 3), but not with plasma sVCAM levels (β = 0.001 [95% CI: −0.005 to 0.008], *P* = .69; and β = 0.006 [95% CI: −0.002 to 0.014], *P* = .13, respectively, Table 3). Additional adjustment for glucose metabolism state (ie NGM, IGM, and T2DM) did not affect the outcomes (data not shown).

**Table 3:** Relationship between nonalcoholic fatty liver disease (NAFLD) susceptibility genes and plasma ALT, triglycerides, sE‐selectin and sVCAM levels in the combined Cohort on Diabetes and Atherosclerosis Maastricht and Hoorn studies

	Effect size of NAFLD risk allele			
	*PNPLA3* (rs738409 G‐allele) (n = 1265)		*GCKR* (rs1260326 T‐allele) (n = 1265)	
	β	95% CI	β	95% CI
Log ALT	0.023	0.005;0.041	0.011	−0.004;0.026
Log Triglycerides	−0.016	−0.029; −0.004	0.017	0.006;0.027
Log sE‐selectin	0.020	0.005;0.036	0.019	0.006;0.032
Log sVCAM	0.006	−0.002;0.014	0.001	−0.005;0.008

Note

Analysed with linear regression, under the assumption of an additive mode of inheritance. Adjusted for age, sex and cohort. Betas represent unstandardized regression coefficients.

## 4 DISCUSSION

Soluble E‐selectin has frequently been used as a plasma biomarker of endothelial dysfunction in epidemiological studies. In the present study, we showed that (a) NAFLD severity, in particular the inflammatory stage, was associated with hepatic E‐selectin expression; (b) E‐selectin mRNA expression in liver, but not in VAT and muscle, was associated with plasma sE‐selectin levels and (c) NAFLD susceptibility genes and liver parenchyma damage (reflected by plasma ALT levels) were associated with plasma sE‐selectin levels, independent of potential confounding factors. Such consistent associations were not observed for sVCAM.

Animal studies have shown that liver sinusoidal endothelial cells are involved in the pathogenesis of NAFLD.^3^ In general, the microvascular endothelium is a major regulator of vasomotor tone, permeability, coagulation, fibrinolysis and smooth muscle cell proliferation. Endothelial dysfunction occurs when one of these functions is impaired. E‐selectin is an adhesion molecule that is specifically expressed on cytokine‐activated endothelial cells. It mediates the adhesion and rolling of leukocytes on the endothelium as a part of the inflammatory response. Shedding of E‐selectin from the damaged and/or cytokine‐activated endothelial cells results in its release (as soluble E‐selectin) into the circulation, which can subsequently be measured as a biomarker of endothelial activation.^33^

Previous experiments have shown that E‐selectin is actively involved in the development of hepatic inflammation in mice exposed to chronic plus binge ethanol feeding.^9^ In the present study, we noted an increased hepatic E‐selectin mRNA expression in parallel to the inflammatory response in LDLR^−/−^ mice fed a Western‐type diet, an established mouse model of rapidly induced NASH.^15^, ^16^ Such a pattern was not observed in plasma, which may be due to the coexistence of systemic inflammation in this mouse model.^34^ A similar relationship between NAFLD severity and hepatic E‐selectin mRNA expression was observed in severely obese individuals undergoing bariatric surgery. Although we did not address which cell type was responsible for the greater hepatic E‐selectin mRNA expression in our studies, for example, hepatocytes, stellate cells, Kupffer cells or endothelial cells, previous immunohistochemical studies have shown that E‐selectin protein was specifically expressed on the vascular endothelium in different inflammatory liver diseases (ie alcoholic fatty liver disease, allograft rejection and primary biliary cirrhosis), but not in normal livers.^35^

In the present study, hepatic E‐selectin mRNA expression, but not VAT or muscle E‐selectin mRNA expression, was associated with plasma sE‐selectin levels. We did not find an association between hepatic VCAM mRNA expression and plasma sVCAM levels. These findings raise the question on whether (the activated endothelium in) NAFLD could be a source of circulating sE‐selectin. Although plasma sE‐selectin was able to discriminate NASH from non‐NASH, the area under the ROC curve of 0.73 is too small to use sE‐selectin as a single, diagnostic biomarker of NASH. Future studies are needed to ascertain whether inclusion of sE‐selectin in a multiple biomarker risk score, for example, in combination with cytokeratin‐18 fragment levels,^36^ can be of clinical value.

In the present study, we corroborated the previously reported, strong relationship between NAFLD and sE‐selectin.^7^, ^8^ In addition, using large cohorts we are able to adjust for potential confounding factors, such as smoking, low‐grade inflammation, plasma lipids, type 2 diabetes and cardiovascular disease, which have all been associated with systemic endothelial dysfunction.^4^, ^5^, ^37^, ^38^, ^39^ We observed that plasma ALT levels were associated with sE‐selectin, independent of these potential confounders. The greatest decrease in the regression coefficient was observed when alcohol, BMI and smoking behaviour were added to the model, which is in contrast to the regression models for sVCAM where inflammatory markers, in particular TNFα, had the greatest effect on the regression coefficient. The independent association of alcohol on sE‐selectin levels might be explained by a direct effect of alcohol on the hepatic endothelium, as previously shown.^9^

The association of NAFLD with sE‐selectin, independent of factors that are associated with systemic endothelial activation, was also demonstrated by the use of common NAFLD susceptibility genes, that is, *PNPLA3* and *GCKR*.^27^ Since individuals are ‘randomized’ at conception to receive an allele that either predisposes to or protects from NAFLD, these gene variants can serve as instruments to make causal inferences about the relationship between NAFLD and sE‐selectin levels. Although pleiotropic effects have been reported for both *PNPLA3* and *GCKR* – which may violate this ‘Mendelian randomization’ assumption^40^; it should be noted that these variants have opposing pleiotropic effects on plasma lipids,^24^, ^29^ and risk of type 2 diabetes and coronary artery disease,^30^, ^31^, ^32^ as summarized in Table S3. As a combination, they therefore serve as a model to disentangle NAFLD from factors that affect systemic endothelial function, despite the presence of pleiotropy. Again, we observed that sE‐selectin levels co‐segregated with plasma ALT levels, not with plasma triglycerides. These associations were not found for sVCAM.

This study has several strengths and limitations. A major strength is the adoption of different methodologies, that is, animal experiments, liver biopsy studies and cohort studies (with both classical biomarkers and the use of distinctive genetic markers), that enabled us to demonstrate that the liver, more specifically NAFLD, is closely linked to circulating sE‐selectin levels. The observational nature of our studies does, however, not allow to exactly determine if and to what extent sE‐selectin is derived from the liver. Other organs besides the inflamed liver can contribute to circulating sE‐selectin as well. In the present study, plasma ALT was used as a liver‐specific biomarker of parenchymal damage. Previous studies have shown that plasma ALT is not a perfect biomarker of NAFLD severity, although simple steatosis and NASH have been associated with greater plasma ALT levels.^41^, ^42^ It is therefore likely that there is a residual ‘NAFLD effect’ on sE‐selectin levels when plasma ALT is entered as an independent variable in the regression model. This phenomenon could explain the significant, independent association of BMI – a strong determinant of NAFLD^43^ – with sE‐selectin levels in our study.

In conclusion, the present study shows that higher expression of hepatic E‐selectin and higher levels of plasma sE‐selectin are associated with NAFLD and related markers. These findings favour further study to elucidate the role of E‐selectin in the pathogenesis of NAFLD and the applicability of sE‐selectin as a plasma biomarker of NAFLD/NASH.

## CONFLICT OF INTEREST

The authors declare that there is no conflict of interest.
