# Supplementary material for: The endothelial function biomarker soluble E‐selectin is associated with nonalcoholic fatty liver disease
Source: Liver Int. 2020 Jan 29;40(5):1079–88. doi: 10.1111/liv.14384 (PMC7317803; doi:10.1111/liv.14384)
Supplement: Supplementary file 1 [file LIV-40-1079-s001.docx]

**Supplementary Materials**

Supplementary Figure 1 – Plasma sE-selectin levels in LDLR^-/-^ mice on a Western-type diet

Supplementary Figure 2 – Receiver operating characteristic curves for sE-selectin levels to discriminate between NASH and the individual NAFLD histological stages in severely obese individuals

Supplementary Table 1 – Primer sequences

Supplementary Table 2 – Determinants of plasma sE-selectin levels in participants of the CODAM study

Supplementary Table 3 – Overview of previously reported associations of *PNPLA3* and *GCKR* with NAFLD and factors that have been associated with systemic endothelial activation

Supplementary References

**Supplementary Figure 1 – Plasma sE-selectin levels in LDLR^-/-^ mice on a Western-type diet**

**
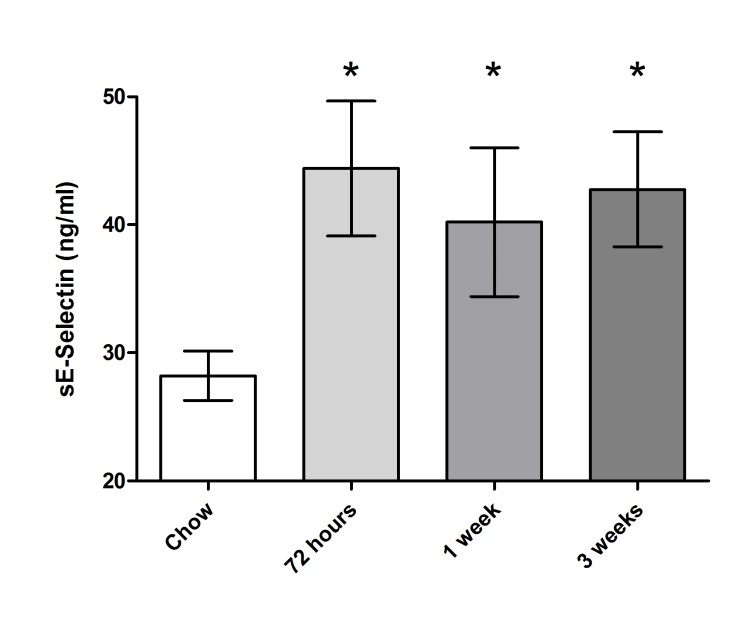
**

Plasma sE-selectin level in LDLR^-/-^ mice fed a Chow diet (n=5) or Western-type diet for 72 hours (n=5), 1 week (n=5), and 3 weeks (n=4). Data are expressed as mean ± SD, analyzed with linear regression. * p < 0.05 versus chow.

**Supplementary Figure 2 – Receiver operating characteristic curves for sE-selectin levels to discriminate between NASH and the individual NAFLD histological stages in severely obese individuals**

**
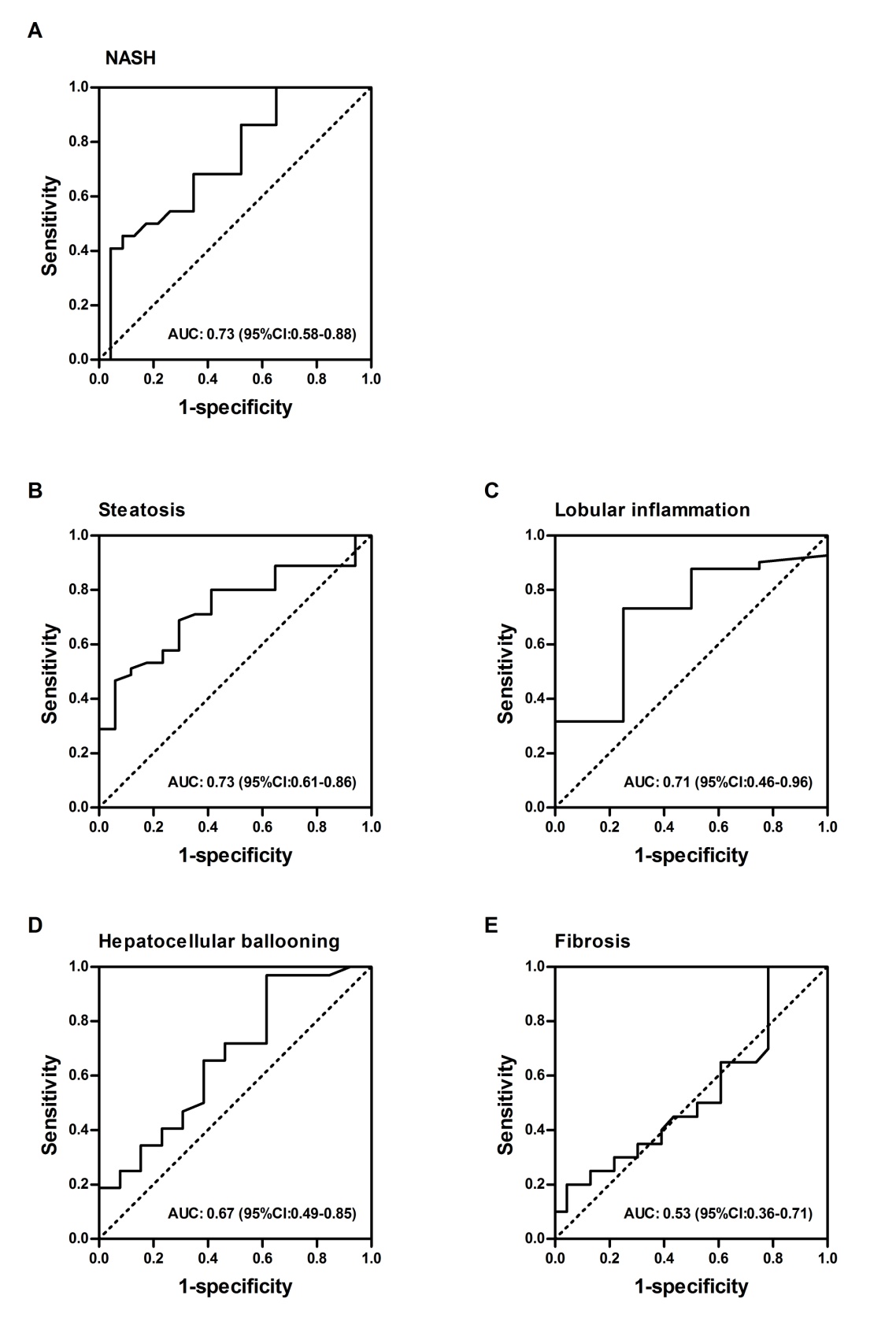
**

Receiver operating characteristic curves for sE-selectin levels to discriminate between NASH (NAS ≥ 5) and no NASH (NAS ≤ 2) (n=45, panel A) and the individual NAFLD histological stages (i.e. steatosis (n=62, panel B), lobular inflammation (n=45, panel C), ballooning (n=45, panel D) and fibrosis (n=43, panel E) in severely obese individuals. Abbreviations: AUC: area under the curve; NAS: nonalcoholic fatty liver disease activity score; NASH: nonalcoholic steatohepatitis; NAFLD: nonalcoholic fatty liver disease.

**Supplementary Table 1 – Primer sequences**

| **Gene** | **FW primer** | **RV primer** |
| --- | --- | --- |
| **TNF** | CATCTTCTCAAAATTCGAGTGACAA | TGGGAGTAGACAAGGTACAACCC |
| **E-selectin** | AGCAGAGTTTCACGTTGCAGG | TGGCGCAGATAAGGCTTCA |
| **Cyclophilin** | TTCCTCCTTTCACAGAATTATTCCA | CCGCCAGTGCCATTATGG |
| **Β2-Micro** | CTTTCTGGTGCTTGTCTCACTGA | GTATGTTCGGCTTCCCATTCTC |

Forward and reverse qPCR primer sequences of all measured genes.

**Supplementary Table 2 – Determinants of plasma sE-selectin levels in participants of the CODAM study**

|  | **CODAM/Hoorn**  **Total population**  **(n = 571)** | |
| --- | --- | --- |
|  | **log plasma sE-selectin** | |
| **Model, independent variables** | **β** | **95% CI** |
| Age | 0.001 | -0.001;0.003 |
| Sex | 0.025 | -0.006;0.056 |
| BMI | 0.005 | 0.001;0.008* |
| Smoking | 0.021 | -0.012;0.053 |
| Alcohol | 0.028 | 0.004;0.052* |
| ALT | 0.249 | 0.160;0.339* |
| Total cholesterol | 0.002 | -0.011;0.015 |
| HDL-cholesterol | -0.225 | -0.450;0.000 |
| Systolic blood pressure | 0.000 | -0.000;0.001 |
| HbA1c | 0.005 | -0.337;0.346 |
| Use of glucose-lowering medication | -0.010 | -0.055;0.035 |
| Use of lipid-modifying medication | 0.002 | -0.033;0.037 |
| C-reactive protein | 0.008 | -0.051;0.067 |
| Amyloid A | 0.004 | -0.076;0.084 |
| Interleukin-6 | 0.018 | -0.077;0.113 |
| Interleukin-8 | 0.099 | 0.009;0.189* |
| Tumor necrosis factor α | -0.023 | -0.137;0.091 |
| History of cardiovascular disease | 0.034 | 0.002;0.066* |

Analyzed with linear regression. Beta’s represent unstandardized regression coefficients. * p < 0.05.

**Supplementary Table 3 – Overview of previously reported associations of *PNPLA3* and *GCKR* with NAFLD and factors that have been associated with systemic endothelial activation**

|  | ***PNPLA3***  **rs738409**  **G allele** | ***GCKR***  **rs1260326**  **T allele** | **Reference** |
| --- | --- | --- | --- |
| **Nonalcoholic fatty liver disease (including NASH)** | ↑ | ↑ | ^1,2^ |
| **Plasma lipids** | ↓ | ↑ | ^3,4^ |
| **Type 2 diabetes** | ↑ | ↓ | ^5^ |
| **Coronary artery disease** | ↓ | ↑ | ^6,7^ |

**References**

1. Romeo S, Kozlitina J, Xing C, et al. Genetic variation in PNPLA3 confers susceptibility to nonalcoholic fatty liver disease. Nature genetics. 2008;40(12):1461-1465.
2. Lin YC, Chang PF, Chang MH, Ni YH. Genetic variants in GCKR and PNPLA3 confer susceptibility to nonalcoholic fatty liver disease in obese individuals. The American journal of clinical nutrition. 2014;99(4):869-874.
3. Vaxillaire M, Cavalcanti-Proenca C, Dechaume A, et al. The common P446L polymorphism in GCKR inversely modulates fasting glucose and triglyceride levels and reduces type 2 diabetes risk in the DESIR prospective general French population. Diabetes. 2008;57(8):2253-2257.
4. Liu DJ, Peloso GM, Yu H, et al. Exome-wide association study of plasma lipids in >300,000 individuals. Nat Genet. 2017;49(12):1758-1766.
5. Mahajan A, Wessel J, Willems SM, et al. Refining the accuracy of validated target identification through coding variant fine-mapping in type 2 diabetes. Nature Genetics. 2018;50(4):559-571.
6. Simons N, Isaacs A, Koek GH, Kuc S, Schaper NC, Brouwers M. PNPLA3, TM6SF2, and MBOAT7 Genotypes and Coronary Artery Disease. Gastroenterology. 2017;152(4):912-913.
7. Simons P, Simons N, Stehouwer CDA, Schalkwijk CG, Schaper NC, Brouwers M. Association of common gene variants in glucokinase regulatory protein with cardiorenal disease: A systematic review and meta-analysis. PloS one. 2018;13(10):e0206174.
